# Weakest students benefit most from a customized educational experience for Generation Y students

**DOI:** 10.7717/peerj.682

**Published:** 2014-12-02

**Authors:** Romesh P. Nalliah, Veerasathpurush Allareddy

**Affiliations:** 1 Office of Dental Education, Harvard School of Dental Medicine, Boston, United States; 2 Harvard School of Public Health, Boston, MA, United States; 3 Department of Orthodontics, College of Dentistry, University of Iowa, Iowa City, IA, United States

**Keywords:** Peer-to-peer learning, Self-directed learning, Critical thinking, Team-based learning, Problem solving, Millennials, Generation Y, Dental education

## Abstract

Most current dental students were born in the 1980s and 1990s and are defined as Generation Y (Gen Y). The authors developed a customized educational experience that brought together some characteristics of Gen Y and the objective of this educational experience was to develop the critical thinking skills of Gen Y students. The objective of the current study is to evaluate outcomes from pre-session and post-session tests. Additionally, we wanted to integrate aspects of team-based learning, self-directed learning and peer-to-peer teaching as a means of reducing the need for intense faculty supervision but maintain positive educational outcomes. Single bitewing x-ray was displayed and informal class discussion was facilitated by a Senior Tutor. A list of questions and concepts that needed to be understood more clearly was made. Student groups self allocated research tasks to members. After conducting research, students presented to class and faculty facilitated discussions aiming to foster critical thinking and identify what information needed to be more thoroughly understood. Pre-session and post-session tests were conducted and compared. Students who scored below 85% in their pre-session test improved their score in the post-session test by a mean of 9.5 points (*p* = 0.02). Those who scored above 95% in their pre-session test scored less in the post-session test (mean reduction of 6.31 points, *p* = 0.001). Findings from this study demonstrate that the weakest students in the class (those who scored below 85% correct in the pre-session test) benefitted most from this unique educational experience.

## Introduction

There is a nationwide shortage of appropriately qualified dental school faculty in the United States (Chmar, Weaver & Valachovic, 2008). A 2001 report by Haden, Weaver & Valachovic (2002) indicated that half of all dental faculty were 50 years of age or older. It is now 13 years since that report was published, and that cohort of faculty will have already retired or are making retirement plans. This loss of experienced faculty is coupled with growth in the number of dental schools and the increasing class sizes in some existing schools. Additionally, it is challenging to attract qualified dentists into dental education and to retain existing dental educators. Some factors suggested for the development of these challenges are the perceived discrepancy in income potential between private practice and full time academia (Nalliah, Howell & Allareddy, 2013) and the increasing student debt (Haden et al., 2000). There is a need for innovative educational curricular that are effective but would require fewer faculty. Additionally, there is a need for team-based learning experiences to match the rapid increase of group practice environments that our students will graduate into (Fox, 2012).

The Oxford Dictionary refers to those born in the 1980s and 1990s as Generation Y (Gen Y) or Millennials (Oxford Dictionary Generation Y). Most current dental school students fit the definition for Gen Y and have several characteristics that are unique to their generation. Research has shown that Gen Y students work well in group environments, multi-task often, are comfortable with technology use and need immediate feedback (Elam, Stratton & Gibson , 2007; Blue, 2009). Educational conditions that cater to these features may create a more effective learning environment for Gen Y students. Our project attempted to bring together all these aspects of teaching and added aspects of learning that the Commission on Dental Accreditation (CODA) has required such as problem-solving and self-directed learning (American Dental Association). Additionally, the intent of Standard 2–10 of the CODA requirements is to increase peer-to-peer learning (American Dental Association) and, therefore, we wanted to include aspects of this in our Gen Y education experience at the Harvard School of Dental Medicine (HSDM). Finally, HSDM strongly believes in Case Method learning and this was the foundation for building a hybrid Problem Based Learning (PBL) curriculum. We built our educational experience on specific clinical cases which served as the starting point for discussion. After building this customized learning experience for Gen Y students in the Harvard School of Dental Medicine, we implemented the educational project with an objective of increasing baseline understanding in diagnostic reasoning and critical thinking while using limited faculty resources. We aimed to move students from “novice behaviors” toward “expert behaviors” (Hendricson et al., 2006) which, we believe, will enable them to develop more rapidly when they enter the patient care phase of their training.

HSDM has four Senior Tutors who are experienced dentists that oversee ten students from each year of dental school and are focused on training them during their clinical years (third and fourth). A Senior Tutor’s role is to oversee the development of diagnostic and treatment planning skills and to enhance the development of critical thinking skills. At the Harvard School of Dental Medicine (HSDM), students spend the first two years at the Harvard Medical School where they are held to the same standards as Harvard medical students. In addition to the full medical curriculum, students take a course at the dental school and, in their last 3 months of second year, they are integrated into the dental school. These students begin intense pre-clinical preparations, lectures and tutorials related to diagnosis, treatment planning, basic restorative dentistry and prevention. The objective of this paper is to describe a One day, Team-Based Learning (TBL) experience for students. The goal of this TBL experience was to introduce aspects of diagnostic reasoning, treatment planning and build critical thinking skills. If successful, by the end of this experience students will improve critical thinking skills and have a higher baseline understanding of how to move from a diagnosis to the development of a patient treatment plan. This may facilitate better learning experiences early in their clinical work as students who are not inhibited by poor critical thinking skills and a poor understanding of the challenges and logistics of treatment planning. From the day after this learning intervention students began a formalized educational curriculum that includes treatment planning and critical thinking. Thus, subsequent measures to determine if the changes in the current intervention were sustained are impossible as many other modes of teaching diagnoses, treatment planning and critical thinking were implemented and the impact of the initial intervention cannot be evaluated. The current paper describes the implementation and reports initial outcomes from the Gen Y education experience at HSDM through pre- and post-session test scores. These tests were implemented to evaluate the educational effectiveness of the teaching intervention. It should be noted that this was a small, quasi-experimental, exploratory study and provides basic pilot data.

## Methods

All data was collected in a rigorous manner to evaluate the educational benefit of this experience. However, it was not initially designed as a research study. Retrospectively, it was determined to analyze and publish this data and approval was provided by Harvard Medical School’s Institutional Review Board.

All data was devoid of student identifiers. Although Harvard University Identification (HUI) numbers were used to pair the pre- and post-tests, the HUI numbers were removed and destroyed once paired. Students were not graded and participation was not mandatory. The study included Doctor of Dental Medicine (DMD) students from the classes of 2014 and 2015. The study was conducted over a two year period on the first day of the 3rd year DMD program.

### Pre-session test

At the start of the first day of 3rd year of DMD program, a pre-session test with questions that evaluate general dental knowledge and tests critical thinking skills was conducted. This test was on paper (rather than computerized) because it was believed a higher participation rate was likely if students could fill the tests immediately after participation and return to the faculty coordinator. There were 21 multiple choice questions relating to clinical situations where students would have to use critical thinking and problem solving skills to determine what to do next. Scores were computed on a 100 point scale and students entered their HUI number to help maintain confidentiality but also allow comparison to the post-session test. Questions were developed by one Senior Tutor and then edited and validated through discussion by all Senior Tutors (the HSDM faculty who focus on training critical thinking skills to pre-doctoral dental students). This was an informal, rather than rigorous, process because this educational experience was not initially planned as a research study. Tests were given in paper form to every student who filled it out and returned it to a box at the front of the classroom.

### Class/Discussion session

A single bitewing x-ray with a carious tooth was displayed to the class and an informal class discussion was facilitated by one Senior Tutor. The facilitated discussion was aimed at identifying what other information a dental provider would need to make a diagnosis, create a problem list, identify what information was needed to make a treatment plan and to determine what concepts were needed to be understood more clearly before proceeding. The discussion lasted about 70 min and at its conclusion students split into groups (HSDM classes are already organized in groups, called societies) and constructed a list of concepts and divided research tasks between the four societies. Society members distributed the investigation topics and conducted research independently using internet resources. Students were encouraged to take notes. Students met as a society and prepared their class presentation over 3 h of independent study time. Then societies worked together to present back to the rest of their class in the presence of three Senior Tutors. Senior Tutors facilitated discussions, asked further questions and endeavored to foster critical thinking during these discussions (in the same way they would when they function one-on-one with students later in the program). The presentations and discussions lasted approximately 75 min and at the conclusion, students completed the post-session test. It should be noted that we did not evaluate results by society.

### Post-session test

The Post-session test was a closed-book test and was identical to the pre-session test. Students entered their Harvard University Identity number to enable comparison to the pre-session test. Post-session test was administered immediately after the group presentations. Students were, again, given a paper copy to fill out immediately and return to a different box (compared to pre-test) at the front of the classroom. There was a 100% return rate.

### Analytical approach

Descriptive statistics were used to examine and summarize the data. The main outcome variables of interest were the pre-session and post-session test scores. Distribution of the outcome variables were examined by using the One-Sample Kolmigorov Smirnov test. Non-parametric tests were used to overcome the requirement of normal distribution. Wilcoxon Signed Rank test was used to examine the paired comparison of pre-session and post-session test scores. The entire sample was divided into four groups based on the distribution of pre-session test scores into: ≤85, >85–90, >90–95, and >95 groups. Within each group, Wilcoxon Signed Rank test was used to examine the paired comparison of pre-session and post-session test scores. All statistical tests were two sided a *p*-value of <0.05 was deemed to be statistically significant. Statistical analyses were conducted using SPSS Version 20.0 software (IBM Inc).

## Results

The study sample comprised of 65 students from two years of DMD classes (Classes of 2014 and 2015). The distribution of the pre-session and post-session test scores are summarized in Table 1. The mean pre-session test score was 90.26 percentage points (standard deviation = 10.39) and the mean post-session test score was 89.96 percentage points (standard deviation = 12.05). The minimum scores in the pre-session and post-session tests were 57.14 and 33 percentage points respectively. The median pre-session and post-session test scores were 90.48 points. Paired sample comparison between the pre-session and post-session test scores showed the scores to be not significantly different (*p* = 0.03).

**Table 1:** Distribution of pre-session and post-session test scores (*N* = 65).

		Pre session test score	Post session test score	*p*-value (Wilcoxon Signed Rank Test)
Mean		90.26	89.96	0.93
Std. deviation		10.39	12.05	
Minimum		57.14	33	
Maximum		100	100	
Percentiles	25	85.71	88.09	
	50	90.48	90.48	
	75	100	100	

Results from the subset analyses within the four pre-session test scoring groups are summarized in Tables 2–5. Overall, in the pre-session test a total of 14 students scored ≤85 points, while 6 students scored >85–90 points, 14 scored >90–95 points, and 31 scored >95 points. Paired sample tests (Wilcoxon Signed Rank) within these four subsets indicated that for students within the ≤85 points group, the post-session test scores increased by an average of 9.5 points (*p* = 0.02). The median scores increased by 14.3 points within this group. There were no significant differences in pre-session and post-session test scores for those within the >85–90 points and >90–95 points groups. For those within the >95 points subgroup, the post-session test scores decreased by an average of 6.31 points (*p* = 0.001). The median scores decreased by 4.76 points within this group.

**Table 2:** Distribution of pre-session and post-session test scores within ≤85 pre-session test group (*N* = 14).

		Pre session test score	Post session test score	*p*-value (Wilcoxon Signed Rank Test)
Mean		73.81	83.33	0.02
Std. deviation		7.64	13.95	
Minimum		57.14	52.38	
Maximum		80.95	100	
Percentiles	25	70.23	77.38	
	50	76.19	90.48	
	75	80.95	91.67	

**Table 3:** Distribution of pre-session and post-session test scores within >85 to ≤90 pre-session test group (*N* = 6).

		Pre session test score	Post session test score	*p*-value (Wilcoxon Signed Rank Test)
Mean		85.71	90.48	0.19
Std. deviation		0	7.97	
Minimum		85.71	80.95	
Maximum		85.71	100	
Percentiles	25	85.71	84.52	
	50	85.71	88.09	
	75	85.71	100	

**Table 4:** Distribution of pre-session and post-session test scores within >90 and ≤95 pre-session test group (*N* = 14).

		Pre session test score	Post session test score	*p*-value (Wilcoxon Signed Rank Test)
Mean		90.48	91.50	0.67
Std. deviation		0	6.78	
Minimum		90.48	76.19	
Maximum		90.48	100	
Percentiles	25	90.48	90.48	
	50	90.48	90.48	
	75	90.48	96.43	

**Table 5:** Distribution of pre-session and post-session test scores within >95 pre-session test group (*N* = 31).

		Pre session test score	Post session test score	*p*-value (Wilcoxon Signed Rank Test)
Mean		98.46	92.15	0.001
Std. deviation		2.26	12.98	
Minimum		95.23	33	
Maximum		100	100	
Percentiles	25	95.23	90.48	
	50	100	95.24	
	75	100	100	

## Discussion

The Harvard School of Dental Medicine (HSDM) has about 35 students per year. Many schools in the United States have class sizes above 100 students and a similar program may not be possible with participation from the whole class. The benefit of a small class size is that the entire class can easily participate in one facilitated discussion. This ensured that results from pre- and post-session tests were related to exactly the same initial discussion.

Senior Tutors are a unique part of the HSDM program. Senior Tutors are dental faculty who must approve every pre-doctoral treatment plan through one-on-one meetings with students where Senior Tutors can challenge every concept that the patient case addresses. However, students who did not receive cases earlier in the 3rd year (due to unsuitable patient cases, failed appointments etc.) tended to be delayed in the development of their knowledge, critical thinking skills and treatment planning skills. This delay kept them a little behind the rest of the class for the whole program and these students often needed remediation in the final months of dental school. One of the objectives of this educational experience was to go through a straightforward case as a class. This would enable students to become familiar with the process of developing and defending their treatment plan with a Senior Tutor and help to improve their knowledge of basic cases which could improve their future learning experiences at HSDM. An unexpected benefit was that weaker students improved their pre- versus post-session test scores the most.

Our educational experience found that the cohort who scored 85% or less in the pre-session test increased their score on the post-session test by a mean of 9.52% (*p* = 0.02). This educational experience was aimed at enhancing diagnostic reasoning, treatment planning and critical thinking skills to enable students to have better educational outcomes throughout the program. Ongoing evaluation of critical thinking skills will evaluate if this early training improved subsequent educational experiences as we had hoped, but the short term outcomes are positive.

### Peer-to-peer teaching

Medical education literature has argued that informal opportunities for medical students to teach are beneficial to learning (Dandavino, Snell & Wiseman, 2007). Our project looked to formalize this process and capitalize on the benefits of peer-to-peer teaching. A systematic review has shown that peer teaching can be effective in clinical healthcare profession education (Secomb, 2008) and research has also shown that peer-to-peer teaching is particularly effective when the teacher is closely relatable for the learner (Ten Cate & Durning, 2007). In our education project the teacher and learners were exactly the same: DMD students on their first day in 3rd year. Each student had researched a specific area and reported back to their team. Students then worked as a team to piece together all the independent components of information to create a presentation that was relevant and answered the questions from the original case.

### Team-based learning

Our Gen Y educational experience also integrated aspects of Team-Based Learning (TBL) as students worked in societies to research, critically appraise information and present information that is relevant to the case. There is strong information suggesting a decline in solo dental practices (American Dental Association, 2006) and a growth in large group practices (Fox, 2012). Gen Y students also enjoy group work (Elam, Stratton & Gibson , 2007) and our educational experience capitalizes on this interest. Expanding these TBL opportunities may also help prepare students for the negotiations and team work necessary to succeed in a group practice.

TBL as an approach could also be a valuable educational tool if learning outcomes remain constant because fewer faculty resources are necessary. Our project was able to demonstrate that TBL did facilitate learning with limited use of faculty resources. There was a total of 70 min (initial class discussion) facilitated by one Senior Tutor, 75 min student presentations and wrap-up facilitated by three Senior Tutors and three hours of independent study. This equates to just below five hours of faculty time. In preceding years, this material was covered in seven hours of introductory lectures by faculty. Introductory lectures did not have aspects of TBL, self study/enquiry or peer-to-peer learning. Standard 3-1 of the CODA guidelines (American Dental Association) requires pre-doctoral programs to have a sufficient number and distribution of faculty and our educational project suggests that learning can happen in an environment that utilizes fewer faculty.

### Self-directed learning

Standard 2–9 of the CODA guidelines requires demonstration of problem solving skills and standard 2–10 requires self-directed learning (American Dental Association). Our educational project touched on both of these skills because students needed to identify shortcomings in their own knowledge, determine a group plan to acquire that knowledge and appraise the different sources of knowledge (which is required in CODA standard 2–21) before relating their findings to the case and making a group presentation.

There was no significant difference between pre- and post- session test scores among those who scored between 85% and 95% in the pre-test. Surprisingly, the cohort that scored 95% or more in their pre-session test actually had a mean reduction in post-session test score by 6.31 (*p* = 0.001). It is possible that those who scored high in the pre-test were just fortuitous in their response. An alternative explanation is that students who began with reasonable knowledge and good critical thinking skills may have second guessed themselves and changed their answers in the post-session test. This could be an important weakness related to the outcome of this educational experience. A part of the reduction in post-session class test scores could also be attributed to ceiling effects (Shadish, Coook & Campbell, 2002) because this cohort of students clustered near the highest score and following class/discussion sessions, the scores could only decrease and not increase (Shadish, Coook & Campbell, 2002). Additionally, the reduction in mean score of those who scored highest in the pre-session test could be related to a weakness in our measuring tool—the post-session tests. However, it is interesting to note that the same group interactions that enabled the students that scored most poorly on the pre-test to have a statistically significant improvement also caused the highest pre-session test scorers to have a statistically significant score reduction.

An alternate explanation could be that the Gen Y education experience had the effect of homogenizing student understanding which is a negative outcome. The Oxford Dictionary defines groupthink as “the practice of thinking or making decisions as a group in a way that discourages creativity or individual responsibility.” (Oxford Dictionary groupthink). Further research with more participants that identify the reasons why students reported changing their correct answers may help to determine if groupthink occurred.

### Self-assessment

The ability to reflect on your performance on the pre-session test and determine if you have shortcomings in knowledge is a form of self-assessment. Standard 2–10 of the CODA guidelines requires graduates to be able to self assess accurately which the cohort who scored over 95% in pre-session failed to do successfully (American Dental Association). A study in 1999 showed that the same metacognitive skills are needed to perform and evaluate the performance on a task and, therefore, the unskilled are also unaware (Kruger & Dunning, 1999). In our study, however, the weakest students showed a statistically significant improvement while the strongest showed a statistically significant decline. It is important to remember our sample included dental students who have already passed rigorous testing in college and graduate school and demonstrated their intelligence to reach this point in their careers. It may be that the findings from the Kruger and Dunning study do not hold true when all included subjects are highly intelligent individuals. Other research has shown that medical students are generally poor self-assessors (Sawdon & Finn, 2014) and dental students may be similar in this respect.

The current study shows short term outcomes of our Gen Y education experience. Evaluation of the students’ ongoing performances in critical thinking, diagnosis and treatment planning will determine the long term value of the session and demonstrate if these short term improvements were sustained.

### Limitations

There are several limitations that we must recognize in evaluating the outcomes of this study. Firstly, this educational experience was implemented in a small school and findings may not be generalizable. Additionally, the program was designed and implemented as a fun way to commence the third year that also served as an introduction to diagnostic reasoning, treatment planning and critical thinking needed to succeed in patient management in the third year of dental school. It was not planned as a rigorous research project. Several specific limitations pertain to the pre-test and post-test. For example, while efforts were made by experts to validate the tests there was no attempts to pilot these questions on non-expert subjects similar to the students we implemented these tests on. Another limitation was that students may have reached the third year with varying experiences in clinical dentistry before dental school. For example, a few students in these classes had worked as dental assistants and they may have had a broader clinical experience to draw from in answering test questions. Therefore, the pre- and post-test may be measuring their recall of these experiences rather than their growth during the educational experience. Finally, on reflection we may have had different findings if we had developed two unique tests to serve as pre- and post-tests rather than implementing the same test twice. Existing knowledge suggests that the pre-test itself may “prime” the students who then know what topics they should focus on during the educational experience which leads to better post-test scores (Richland, Kornell & Kao, 2009). However, since we wanted our students to learn from this experience if the pre-test “primed” them it is a strength of the experience even though it is a weakness of the study.

Although this educational intervention was not planned as a research study, rigorous processes were implemented to measure the educational value of the intervention. The current study is a retrospective, descriptive study that capitalizes on the standardized, high quality data that had been collected for educational purpose.

## Conclusions

This study describes an educational experience that was custom-made for Gen Y students. The project integrated aspects of peer-to-peer learning, self-directed learning, team-based learning, problem solving, appraising literature and dental information. The entire experience was case based and involved immediate feedback to students. Our retrospective analysis of pre- and post-session tests found a statistically significant improvement in performance of those who scored below 85% on the pre-session test and a decline in those who scored above 95% on the pre-session test.

## Supplemental Information

10.7717/peerj.682/supp-1 Data S1 Raw data

10.7717/peerj.682/supp-2 Table S1 Distribution of pre-session and post-session test scores (*N* = 65)

10.7717/peerj.682/supp-3 Table S2 Distribution of pre-session and post-session test scores within ≤85 pre-session test group (*N* = 14)

10.7717/peerj.682/supp-4 Table S3 Distribution of pre-session and post-session test scores within >85 to ≤90 pre-session test group (*N* = 6)

10.7717/peerj.682/supp-5 Table S4 Distribution of pre-session and post-session test scores within >90 and ≤95 pre-session test group (*N* = 14)

10.7717/peerj.682/supp-6 Table S5 Distribution of pre-session and post-session test scores within >95 pre-session test group (*N* = 31)
