# Supplementary material for: Weakest students benefit most from a customized educational experience for Generation Y students
Source: PeerJ. 2014 Dec 2;2:e682. doi: 10.7717/peerj.682 (PMC4260125; doi:10.7717/peerj.682)
Supplement: Data S1 [file peerj-02-682-s001.pdf]

Q1_Pretest	Q2_Pretest	Q3_Pretest	Q4_Pretest	Total_Pretest	Pretest_Per		Q1_Posttest	Q2_Posttest	Q3_Posttest	Q4_Posttest
5	6	1	4	16	76.1904762		5	5	5	4
5	5	5	4	19	90.4761905		5	5	5	4
6	6	5	4	21	100		6	6	5	4
5	5	5	4	19	90.4761905		6	6	5	4
6	6	5	4	21	100		5	5	5	4
4	5	5	4	18	85.7142857		4	4	5	4
4	6	1	4	15	71.4285714		6	5	5	4
5	6	5	4	20	95.2380952		6	5	5	4
5	5	5	4	19	90.4761905		5	5	5	4
6	6	5	4	21	100		5	5	5	4
5	5	5	4	19	90.4761905		6	6	5	4
4	4	5	4	17	80.952381		4	4	5	4
6	5	5	4	20	95.2380952		5	5	5	4
6	6	5	4	21	100		6	6	5	4
6	6	5	4	21	100		5	5	5	4
4	5	5	4	18	85.7142857		4	5	5	4
6	6	5	4	21	100		6	6	5	4
5	4	5	4	18	85.7142857		6	4	5	4
6	6	5	4	21	100		6	6	5	4
5	5	5	4	19	90.4761905		5	5	5	4
4	4	5	4	17	80.952381		4	4	5	4
5	5	1	4	15	71.4285714		5	5	1	0
5	5	5	4	19	90.4761905		5	5	5	4
5	5	5	4	19	90.4761905		6	6	5	4
4	6	5	4	19	90.4761905		5	5	5	4
6	6	5	4	21	100		6	6	5	4

Total_Posttes	Posttest_Per
19	90.4761905
19	90.4761905
21	100
21	100
19	90.4761905
17	80.952381
20	95.2380952
20	95.2380952
19	90.4761905
19	90.4761905
21	100
17	80.952381
19	90.4761905
21	100
19	90.4761905
18	85.7142857
21	100
19	90.4761905
21	100
19	90.4761905
17	80.952381
11	52.3809524
19	90.4761905
21	100
19	90.4761905
21	100

4	6	5	4	19	90.4761905		4	6	5	4
6	6	0	4	16	76.1904762		6	6	5	4
6	6	5	4	21	100		4	5	5	4
4	5	1	4	14	66.6666667		6	5	5	4
6	6	5	4	21	100		6	6	5	4
6	6	5	4	21	100		6	6	5	4
6	6	5	4	21	100		5	6	5	4
5	6	5	4	20	95.2380952		5	5	5	4
6	6	5	4	21	100		4	5	5	4
5	6	5	4	20	95.2380952		6	6	5	4
5	4	5	4	18	85.7142857		6	6	5	4
5	5	5	2	17	80.952381		5	5	5	4
4	4	5	2	15	71.4285714		4	6	5	4
6	6	5	4	21	100		5	5	5	4
2	3	5	2	12	57.1428571		2	3	5	4
6	5	5	4	20	95.2380952		5	5	5	2
6	4	5	2	17	80.952381		6	6	5	2
4	6	5	4	19	90.4761905		4	5	5	2
6	5	5	4	20	95.2380952		6	6	5	4
6	6	5	4	21	100		6	6	5	4
4	6	5	4	19	90.4761905		4	6	5	4
6	6	5	4	21	100		6	6	5	4
3	4	5	4	16	76.1904762		5	5	5	2
5	6	5	4	20	95.2380952		5	5	5	4
5	6	5	4	20	95.2380952		5	5	4	1
5	6	5	4	20	95.2380952		6	5	5	4
4	6	5	4	19	90.4761905		4	4	5	4

19	90.4761905
21	100
18	85.7142857
20	95.2380952
21	100
21	100
20	95.2380952
19	90.4761905
18	85.7142857
21	100
21	100
19	90.4761905
19	90.4761905
19	90.4761905
14	66.6666667
17	80.952381
19	90.4761905
16	76.1904762
21	100
21	100
19	90.4761905
21	100
17	80.952381
19	90.4761905
15	71.4285714
20	95.2380952
17	80.952381

6	6	5	4	21	100		6	6	5	4
6	6	5	4	21	100		6	6	5	4
5	5	3	4	17	80.952381		5	5	5	4
4	6	5	4	19	90.4761905		6	5	5	4
4	5	5	4	18	85.7142857		4	5	5	4
5	5	5	4	19	90.4761905		6	5	5	4
6	6	5	4	21	100		6	6	5	4
6	6	5	4	21	100		5	5	5	4
5	1	5	2	13	61.9047619		5	1	5	2
4	5	5	4	18	85.7142857		6	6	5	4
6	6	5	4	21	100		5	5	5	4
5	6	5	4	20	95.2380952		5	5	5	4

21	100
21	100
19	90.4761905
20	95.2380952
18	85.7142857
20	95.2380952
21	100
19	90.4761905
13	61.9047619
21	100
19	90.4761905
19	33
