# Supplementary material for: Weakest students benefit most from a customized educational experience for Generation Y students
Source: PeerJ. 2014 Dec 2;2:e682. doi: 10.7717/peerj.682 (PMC4260125; doi:10.7717/peerj.682)
Supplement: Table S1 [file peerj-02-682-s002.pdf]

Table 1. Distribution of pre-session and post-session test scores (N = 65)

		Pre session test score	Post session test score	p-value (Wilcoxon Signed Rank Test)
Mean		90.26	89.96	0.93
Std. Deviation		10.39	12.05	
Minimum		57.14	33	
Maximum		100	100	
Percentiles	25	85.71	88.09	
	50	90.48	90.48	
	75	100	100	
