# Supplementary material for: Weakest students benefit most from a customized educational experience for Generation Y students
Source: PeerJ. 2014 Dec 2;2:e682. doi: 10.7717/peerj.682 (PMC4260125; doi:10.7717/peerj.682)
Supplement: Table S2 [file peerj-02-682-s003.pdf]

Table 2. Distribution of pre-session and post-session test scores within <=85 pre-session test group (N = 14)

		Pre session test score	Post session test score	p-value (Wilcoxon Signed Rank Test)
Mean		73.81	83.33	0.02
Std. Deviation		7.64	13.95	
Minimum		57.14	52.38	
Maximum		80.95	100	
Percentiles	25	70.23	77.38	
	50	76.19	90.48	
	75	80.95	91.67	
