# Supplementary material for: Weakest students benefit most from a customized educational experience for Generation Y students
Source: PeerJ. 2014 Dec 2;2:e682. doi: 10.7717/peerj.682 (PMC4260125; doi:10.7717/peerj.682)
Supplement: Table S3 [file peerj-02-682-s004.pdf]

Table 3. Distribution of pre-session and post-session test scores within >85 to <=90 pre-session test group (N = 6)

		Pre session test score	Post session test score	p-value (Wilcoxon Signed Rank Test)
Mean		85.71	90.48	0.19
Std. Deviation		0	7.97	
Minimum		85.71	80.95	
Maximum		85.71	100	
Percentiles	25	85.71	84.52	
	50	85.71	88.09	
	75	85.71	100	
