# Supplementary material for: Weakest students benefit most from a customized educational experience for Generation Y students
Source: PeerJ. 2014 Dec 2;2:e682. doi: 10.7717/peerj.682 (PMC4260125; doi:10.7717/peerj.682)
Supplement: Table S4 [file peerj-02-682-s005.pdf]

Table 4. Distribution of pre-session and post-session test scores within >90 and <=95 pre-session test group (N = 14)

		Pre session test score	Post session test score	p-value (Wilcoxon Signed Rank Test)
Mean		90.48	91.50	0.67
Std. Deviation		0	6.78	
Minimum		90.48	76.19	
Maximum		90.48	100	
	25	90.48	90.48	
Percentiles	50	90.48	90.48	
	75	90.48	96.43	
