# Supplementary material for: Weakest students benefit most from a customized educational experience for Generation Y students
Source: PeerJ. 2014 Dec 2;2:e682. doi: 10.7717/peerj.682 (PMC4260125; doi:10.7717/peerj.682)
Supplement: Table S5 [file peerj-02-682-s006.pdf]

Table 5. Distribution of pre-session and post-session test scores within >95 pre-session test group (N = 31)

		Pre session test score	Post session test score	p-value (Wilcoxon Signed Rank Test)
Mean		98.46	92.15	0.001
Std. Deviation		2.26	12.98	
Minimum		95.23	33	
Maximum		100	100	
	25	95.23	90.48	
Percentiles	50	100	95.24	
	75	100	100	
